# Inactivation of KDM6A promotes the progression of colorectal cancer by enhancing the glycolysis

**DOI:** 10.1186/s40001-024-01828-1

**Published:** 2024-06-06

**Authors:** Dexiang Zhang, Xiaohong Zhao, Yu Gao, Meixing Wang, Mi Xiao, Kaihua Zhu, Wei Niu, Yuedi Dai

**Affiliations:** 1 General Surgery Department, Zhongshan-Xuhui Hospital Affiliated to Fudan University, 966th, Middle Huaihai Road, Shanghai, 200031 China; 2 Women Health Care Department, Hainan Women and Children Medical Center, Haikou, 570312 Hainan China; 3 Department of Medical Oncology, Cancer Hospital of Fudan University, Minhang, 106th, Ruili Rd., Shanghai, 200240 China

**Keywords:** KDM6A, Colorectal cancer, LDHA, Cancer metabolism

## Abstract

KDM6A (lysine demethylase 6A) has been reported to undergo inactivating mutations in colorectal cancer, but its function in the progression of colorectal cancer has not been evaluated using animal models of colorectal cancer. In this study, we found that knocking out KDM6A expression in mouse intestinal epithelium increased the length of villus and crypt, promoting the development of AOM (azoxymethane)/DSS (dextran sulfate sodium salt)-induced colorectal cancer. On the other hand, knocking down KDM6A expression promoted the growth of colorectal cancer cells. In molecular mechanism studies, we found that KDM6A interacts with HIF-1α; knocking down KDM6A promotes the binding of HIF-1α to the LDHA promoter, thereby promoting LDHA expression and lactate production, enhancing glycolysis. Knocking down LDHA reversed the malignant phenotype caused by KDM6A expression loss. In summary, this study using animal models revealed that KDM6A loss promotes the progression of colorectal cancer through reprogramming the metabolism of the colorectal cancer cells, suggesting that restoring the function of KDM6A is likely to be one of the strategies for colorectal cancer treatment.

**Supplementary Information:**

The online version contains supplementary material available at 10.1186/s40001-024-01828-1.

## Introduction

KDM6A, also known as UTX, belongs to KDM6 Jumonji histone demethylase subfamily, and it can remove mono-, di- and tri-methyl groups from methylated lysine 27 of histone H3 (H3K27me1/2/3) [1]. Insights into how KDM6A mutations affect the epigenetic landscape to promote carcinogenesis could help reveal potential new treatment approaches.

The function of KDM6A in tumors has sporadically been reported. KDM6A loss triggers an epigenetic switch that disrupts urothelial differentiation and induces a neoplastic state characterized by increased cell proliferation in bladder cancer [2–4]. Disruption of KDM6A is one of the most common somatic alternations in bladder cancer [5, 6]. In the bladder cancer with higher frequency of KDM6A inactivation mutation, increased KI67 labeling index, upregulated glycolysis, DNA repair, mTORC1 signaling, features of the unfolded protein response, and altered cholesterol homeostasis were observed [6].

Currently, KDM6A has been reported to regulate gene expression through both methylation-dependent and methylation-independent mechanisms [7, 8]. KDM6A is upregulated in gastric cancer and can regulate the expression of SALL4, thereby promoting the growth and metastasis of gastric cancer [9]. In renal cell carcinoma, KDM6A can upregulate the expression of autophagy-related genes [10]. In pancreatic cancer, loss of KDM6A leads to upregulation of CXCL1 expression, altering the immune microenvironment and recruiting tumor-associated neutrophils [11]. In pancreatic cancer, KDM6A inactivation leads to activation of super-enhancers, regulating tumor plasticity and making tumor cells more sensitive to BET inhibitors [12]. The inactivation of KDM6A triggered an alveolar-like lineage conversion of basal mammary epithelial cells and accelerated formation of luminal-like tumors [13, 14].

It has been reported that colorectal cancer (CRC) with lymph node metastasis has a higher frequency of KDM6A mutations [15]. Inactivation of KDM6A leads to changes in H3K27 methylation, increasing the stemness of colorectal cancer stem cells and promoting chemoresistance [16]. However, some reports claim that higher expression of KDM6A predicts the advanced colorectal cancer. High expression of KDM6A is associated with mismatch repair status (proficient or deficient), and elevated KDM6A expression and pMMR status indicate poor prognosis [17]. In addition, studies have shown that inhibiting the demethylase activity of KDM6A can eliminate colorectal cancer tumor-initiating cells and suppress tumor development [18]. Moreover, oxaliplatin significantly upregulates KDM6A expression and reduces H3K27 methylation, and inhibiting KDM6A expression enhances the efficacy of oxaliplatin [16]. It seems that the functions of KDM6A in colorectal cancer have been controversial, and further investigation is urgently needed to clarify its role in colorectal cancer.

In this study, we utilized a conditional knockout mouse model of KDM6A to investigate its function in colorectal cancer, and explored the underlying mechanisms.

## Materials and methods

### Cell culture

Colorectal cancer cells (HCT116, RKO, HCT8, HT29) and normal epithelia cell line (NCM460) were obtained from the Cell Bank, Chinese Academy of Sciences. Cells were cultured in DMEM. Fetal bovine serum (FBS, 10%) and antibiotics (100 U/ml penicillin and 100 µg/ml streptomycin) were added to all media. All cells were cultured in a constant temperature incubator (5% CO_2_, 37 °C). Cell transfection was performed using Lipofectamine 8000 according to the instructions.

### Immunohistochemistry

After dewaxing and rehydration of tissue sections, they were placed in EDTA solution for high-temperature retrieval for 30 min. After natural cooling to room temperature, endogenous peroxidase inhibitor was used to block endogenous peroxidase for 15 min. The tissue sections were washed with PBS for 1–2 times, and then incubated with Sox9 antibody (CST, #34330, 1:500) and Ki67 antibody (proteintech, 27309-1-AP, 1:8000) at 4 °C overnight. Then, the tissues were washed with PBS, and incubated with secondary antibody at room temperature for 1 h. 3,3ʹ-diaminobenzidine (DAB) was used to develop the immunohistochemical signals. All tissue sections were counterstained with hematoxylin, and the staining intensity and protein expression level were automatically scored by the Vectra2 system.

### HE staining

After dewaxing and rehydration of tissue sections, the slices were put into hematoxylin staining solution for 3 min. After rinsing with water and differentiation with 1% hydrochloric acid alcohol for a few seconds, the tissues were then counterstained with 0.6% ammonia water for bluing. Finally, the tissues were put into eosin staining solution for 1–3 min. The slices were dried, dehydrated, and then mounted for photography under a microscope.

### The mouse model induced by AOM/DSS

Pathogen-free 12-week-old male KDM6A^f/f^ mice or Villin-Cre; KDM6A ^f/f^ mice on a BALB/c genetic background were housed under SPF conditions with free access to food and water during the experiments. Mice were injected intraperitoneally with AOM (12 mg/kg body weight) dissolved in physiological saline. After a five-day interval, the mice were then administered 2% DSS through their drinking water for a duration of five days. Following this, regular water was provided for a period of 16 days. This cycle was repeated twice to ensure consistent exposure to AOM and DSS. Animals were killed at the indicated time point for histological analysis. All animal experiments were conducted in accordance with the guidelines for the care and use of laboratory animals set by the Committee for Animal Experimentation of Hainan Medical University, and the protocols were approved by the committee.

### Western blot

Cells were washed twice with PBS and then lysed on ice using RIPA lysis buffer containing protease and phosphatase inhibitors. The cell lysate was centrifuged and the supernatant was collected. The protein concentration was quantified using the BCA protein assay kit. Equal amounts of protein were loaded onto an SDS-PAGE gel for electrophoresis. After protein separation, the proteins were transferred onto a PVDF membrane. The membrane was subsequently incubated with specific primary antibodies overnight at 4 °C, and then incubated with HRP-conjugated secondary antibodies for 1–2 h. The immunosignals were detected using a chemiluminescent substrate (Millipore, WBKLS0050) and analyzed using Image Lab software. The primary antibodies used in this experiment included: KDM6A (proteintech, 23984-1-AP, 1:1000), tubulin (Santa Cruz Biotechnolog, sc-5286, 1:4000), LDHA (proteintech, 19987-1-AP, 1:1000), Flag (proteintech, 66008-4-lG, 1:1000), and HIF-1α (Cell signaling technology, #36169, 1:1000) (Additional file 1).

### CCK8 assay

Cells were seeded at a density of 1 × 10^3^ cells per well in a 96-well plate and incubated in a constant temperature incubator set at 37 °C with 5% CO_2_. The next day, the old medium was replaced with fresh medium containing 10% CCK8, and the plate was incubated in the incubator for 2 h. The absorbance at 450 nm was measured daily starting from the initiation of the experiment.

### Colony formation assay for the soft agar

Cells were seeded in a 24-well plate, and the following day, the cell confluence reached approximately 50%. Subsequently, the cells were digested and a cell suspension was prepared. The lower agar layer was prepared according to the following formula: 20% FBS, 40% 2 × RPMI1640 (Basal Medium Eagle), 40% agar (1.25% w/v). 400 μl of the mixture was added to each well, and the plate was then placed in a 37 °C incubator until the gel solidified. The upper agar layer was prepared and mixed evenly with the cell suspension. 400 μl (1 × 10^3^ cells) was added to each well and placed in a constant temperature incubator (37 °C, 5% CO_2_) for 10–14 days. The formula for the upper agar layer was: 25% FBS, 37.5% 2 × RPMI1640, 37.5% agar (1.0% w/v), 0.8% 2 mM l-glutamine. Five random fields were selected under a microscope for clone counting.

### EdU assay

Cells were seeded in a 96-well plate at a density of 2 × 104 cells per well. The Cell-Light EdU Apollo567 In Vitro Kit (Ruibo Biotechnology, C10310-1) was used for detection. Images were captured and analyzed using a high-resolution fluorescence microscope.

### Measurement of the lactate content

Lactate production in the medium was detected by using Enzychrom l-Lactate Assay Kit. Results were normalized to the total protein concentration.

### Knockdown of KDM6A and LDHA

shRNA targeting KDM6A and LDHA was designed using the Sigma website, and clone them into the pLKO.1-puro vector. The lentivirus was packaged using 293T cells, with packaging vectors psPAX2 and pMD2.G. Then, the viral supernatant was collected, concentrates using PEG8000 at 4 °C and 1600*g* for 1 h, removed the supernatant, and dissolved in 2 ml DMEM. Cells with 50%–60% confluency in a 6-well plate were carefully selected for the experiment. To introduce the lentivirus, 400 µl of the lentivirus was added to the cells and incubated overnight. After two days, the cells were further treated with puromycin (1 mg/ml) for a duration of 4 days to facilitate the selection of stable cell lines. To assess the expression levels of KDM6A and LDHA, Western blot analysis was performed. This technique allowed for the detection and quantification of the proteins of interest in the selected cell lines.

### Immunoprecipitation

Immunoprecipitation (IP) is used to detect the interaction between endogenously expressed proteins. After treating the cells, they are lysed using an IP lysis buffer containing proteinase and phosphatase inhibitors. After centrifugation, the lysate is separated and the supernatant is carefully collected. Subsequently, 1 μg of antibody is introduced and left to incubate with the supernatant overnight at a temperature of 4 °C. On the following day, 40 μl of Protein A/G beads (bimake.com, B23202) are added and allowed to incubate for 4 h at 4 °C. The beads are then washed three times with wash buffer before 1× loading buffer is added for subsequent Western blotting analysis.

### ChIP

Cells were seeded in a 10-cm dish. When the confluency reached 90%, the cells were cross-linked with 1% formaldehyde for 10 min and incubated with 125 mM glycine at room temperature for 3 min. The cells underwent two washes with pre-chilled PBS. Subsequently, the cells were gathered in 2 ml of DTT solution (100 mM pH 9.5 Tris–HCl, 10 mM DTT) and left to incubate at room temperature for 10 min. Afterward, the cells were centrifuged at 4 °C and 5000*g* for 5 min. The resulting cell pellet was then suspended in 150 μl of SDS lysis buffer (50 mM Tris–HCl at pH 8.0, 2 mM EDTA, and 1% SDS) containing protease and phosphatase inhibitors. DNA fragments were sheared to an average size of approximately 500 bp using a 12% power ultrasonic sonicator, and the shearing was performed according to the ChIP-IT Express shearing Kit (Active motif, 53008). qPCR was used to analyze the binding of HIF-1α to the LDHA promoter.

## Results

### Knocking out KDM6A leads to elongation of small intestinal villi, increased crypt length, and enhanced cell proliferation

**Fig. 1:**
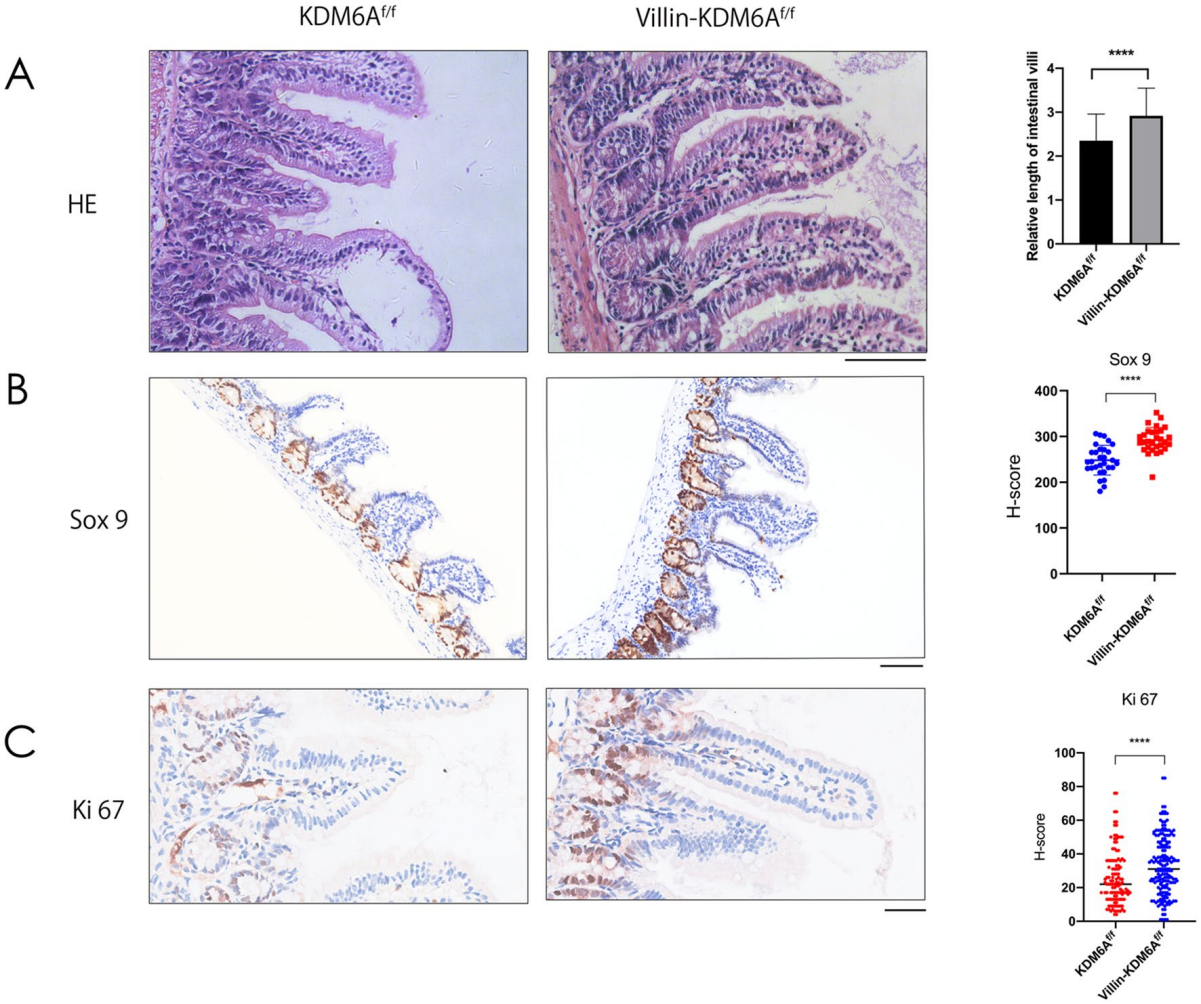
Knocking out KDM6A results the elongation of small intestinal villi and crypts, and the increased cell proliferation. **A** The length of small intestinal villi was measured and analyzed using HE staining. Scale bar: 50 µm. **B** The expression of SOX9 in small intestinal epithelium was detected and analyzed using immunohistochemistry. Scale bar: 50 µm. **C** The expression of Ki67 in small intestinal epithelium was detected and analyzed using immunohistochemistry. Scale bar: 25 µm. ****, *P* < 0.0001

To study the role of KDM6A in maintaining normal intestinal epithelial homeostasis, Villin-Cre mice were crossed with KDM6A^f/f^ mice to delete KDM6A expression in the mouse intestinal epithelium. HE staining showed that after KDM6A knockout, the length of small intestinal villi as well as the expression of crypt marker SOX9 increased (Fig. 1A, B). Additionally, the expression of the cell proliferation marker Ki67 increased in the intestinal epithelial cells after KDM6A knockout (Fig. 1C). These findings suggest that KDM6A expression plays an important role in maintaining intestinal epithelial homeostasis.

### Knocking out KDM6A promotes the occurrence of colorectal cancer

**Fig. 2:**
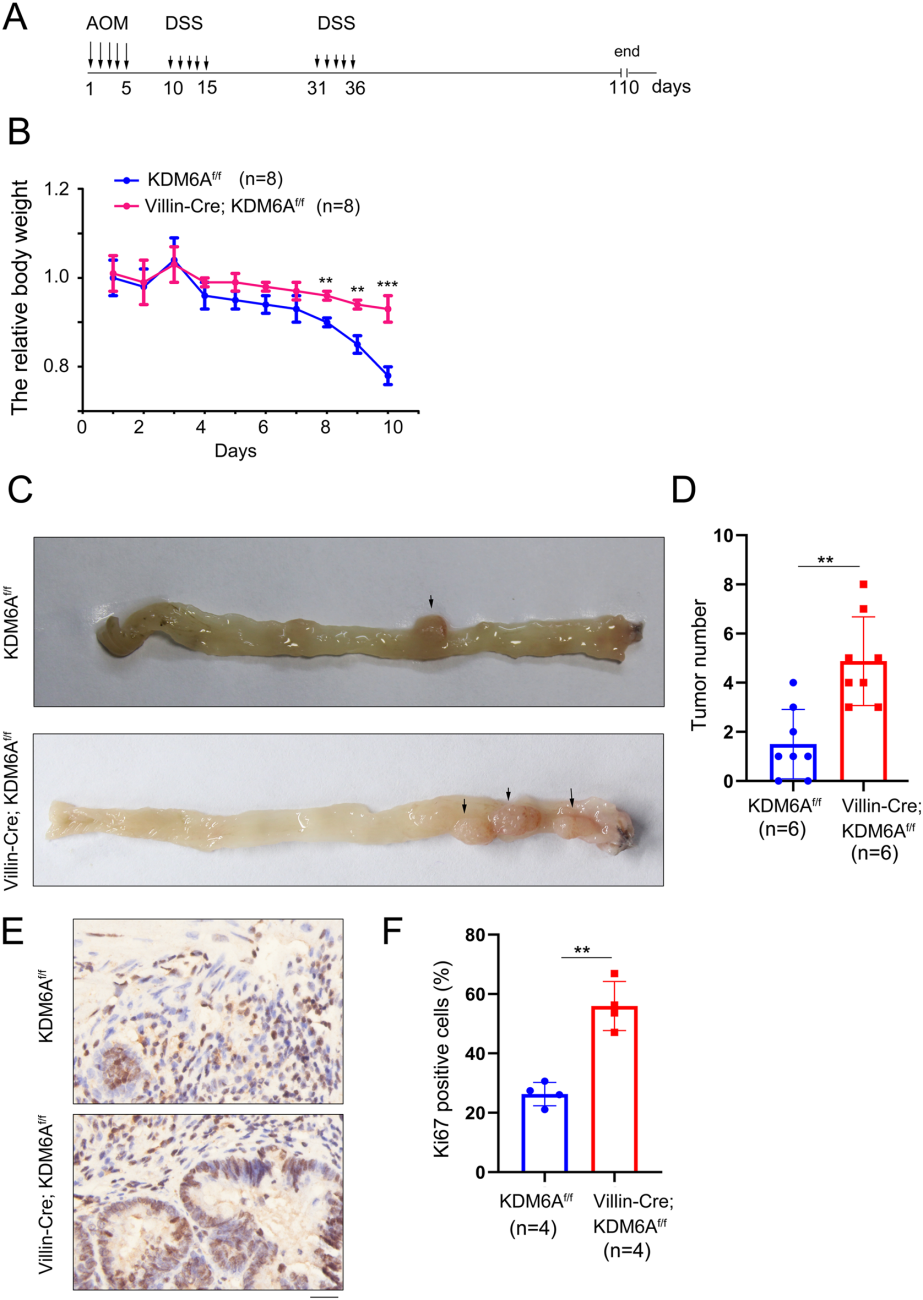
Knocking out KDM6A promotes the occurrence of colorectal cancer. **A** Schematic diagram of AOM/DSS treatment. **B** the body weight of the mice upon the treatment of AOM/DSS. **C**, **D** Representative images of tumor formed in control mice and KDM6A knockout mice, the tumors were counted and the statistical analysis was performed. **E, F** Immunohistochemical staining was performed to examine the expression of Ki67 in tumor tissues formed in control mice and KDM6A knockout mice, and the statistical analysis of Ki67-positive cells was performed. Scale bar: 25 µm. **, *P* < 0.01

In order to study the function of KDM6A in the development of colorectal cancer, we used AOM/DSS treatment to induce colorectal cancer (Fig. 2A). Compared to the mice with the deletion of KDM6A, the body weight of the control mice was more sensitive to the treatment (Fig. 2B). After knocking out the expression of KDM6A, more tumors were formed in the colon of mice (Fig. 2C, D). Furthermore, the tumor tissues with KDM6A knocked-out expression exhibited elevated Ki67 expression, as evidenced by Ki67 staining (Fig. 2E, F).

### Knockdown of KDM6A expression enhances the proliferation of colorectal cancer cells

**Fig. 3:**
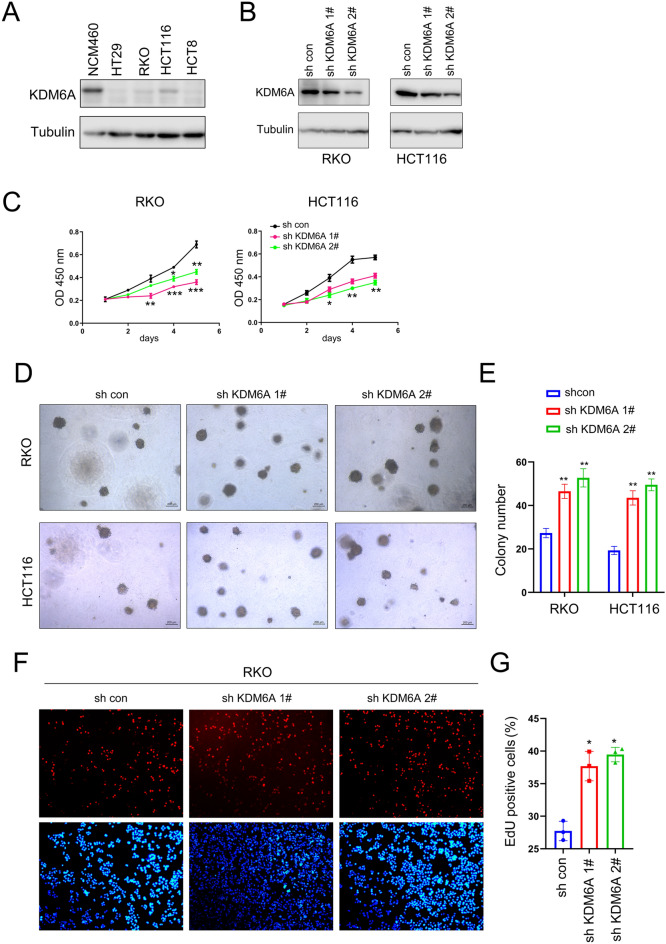
Knocking down KDM6A expression promotes the growth of colorectal cancer cells. **A** The protein levels of KDM6A in the normal cell line NCM460 and colorectal cancer cell lines were examined using Western blot. **B** Western blot was performed to examine the knock down of KDM6A in HCT116 and RKO cells. **C** The growth of colorectal cells was assessed using the CCK8 assay to investigate the impact of KDM6A knockdown. **D**, **E** The effect of KDM6A knockdown on the anchorage-independent growth of colorectal cells was evaluated through the soft agar colony formation assay. Scale bar, 200 µm. **F**, **G** EdU assay was performed to detect the effect of KDM6A knockdown on the proliferation of colorectal cells. *, *P* < 0.05; **, *P* < 0.01; ***, *P* < 0.001

Next, we examined the protein levels of KDM6A in normal epithelial cells (NCM460) and colorectal cancer cell lines. It was found that the protein levels of KDM6A was downregulated in colorectal cancer cell lines (Fig. 3A). Then, we knocked down KDM6A expression in colorectal cancer cell lines and used CCK8 assay, soft agar colony formation assay, and EdU assay to detect the effects of KDM6A knockdown on the growth and proliferation of colorectal cancer cells (Fig. 3B). The results showed that knocking down KDM6A expression promoted the growth of colorectal cells in liquid culture medium and enhanced the colony formation ability of colorectal cancer cells on soft agar (Fig. 3C–E). Moreover, knocking down KDM6A expression promoted the proliferation of colorectal cells (Fig. 3F, G). This suggests that knocking down KDM6A expression promotes the malignant phenotype of colorectal cells.

### Knockdown of KDM6A upregulates LDHA expression, promoting glycolysis

**Fig. 4:**
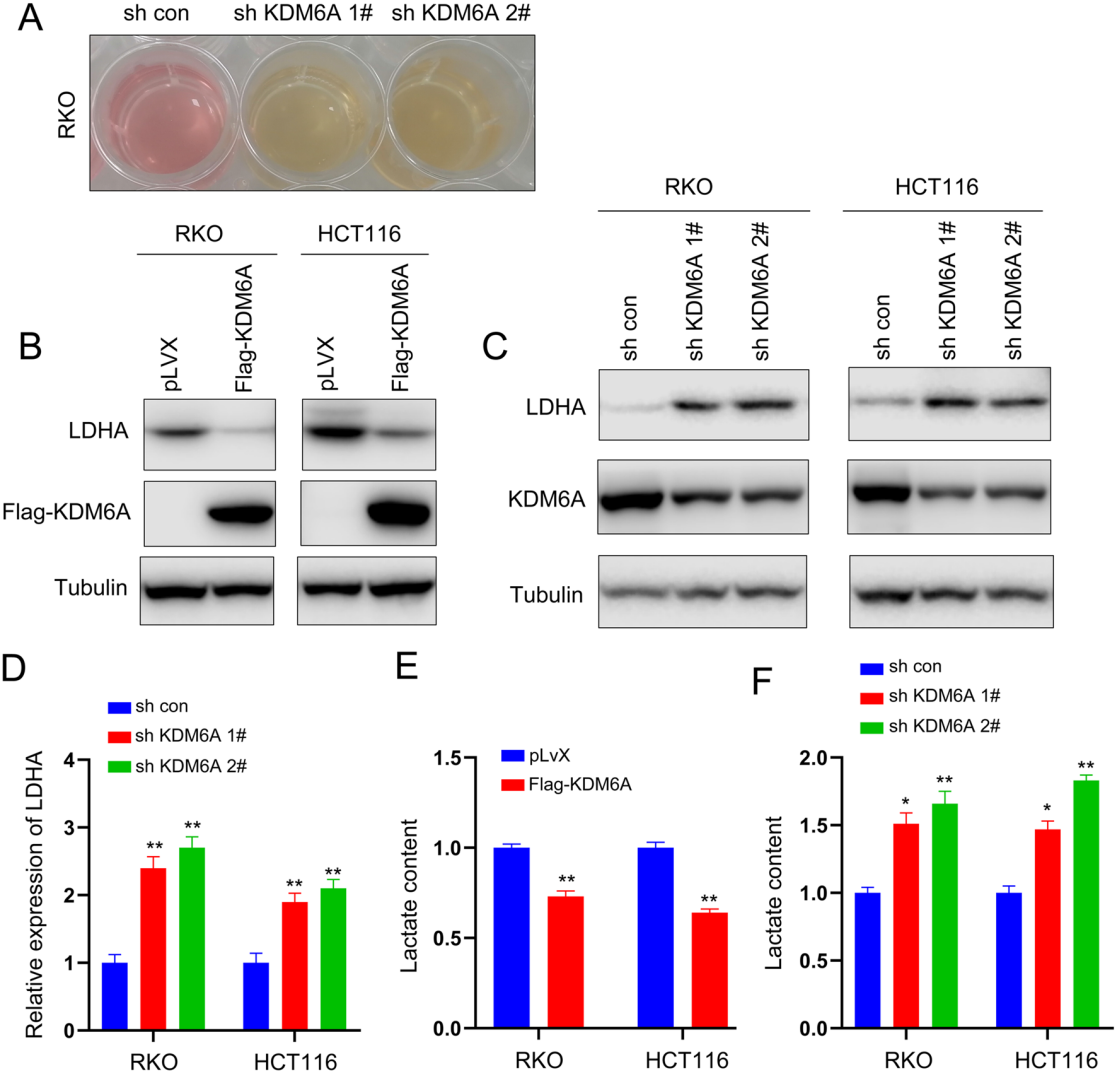
Knockdown of KDM6A promotes glycolysis. **A** Color of the medium incubated with the control cells and colorectal cancer cells knocking down KDM6A expression. **B**, **C** Western blot analysis was performed to examine the effect of overexpressing or knocking down KDM6A on LDHA protein levels. **D** qPCR analysis was performed to examine the effect of knocking down KDM6A on LDHA mRNA levels. **E**, **F** The effect of overexpressing or knocking down KDM6A on lactate levels was examined. *, *P* < 0.05; **, *P* < 0.01

During cell culture, we observed that the culture medium of cells with knocked down KDM6A expression turned yellow (Fig. 4A), indicating increased acid production in colorectal cancer cells after KDM6A knockdown. Some studies have suggested that the production of lactate from glycolysis leads to yellowing of the cell culture medium [19]. Therefore, we first examined the expression of glycolysis-related enzymes. The results showed that overexpression of KDM6A inhibited LDHA expression (Fig. 4B), while knockdown of KDM6A upregulated both the protein and mRNA levels of LDHA (Fig. 4C, D). Next, we analyzed the impact of KDM6A expression on lactate production. Overexpression of KDM6A reduced lactate levels in colorectal cancer cells, while knockdown of KDM6A increased lactate levels (Fig. 4E, F).

### KDM6A inhibits the binding of HIF1α to the LDHA promoter

**Fig. 5:**
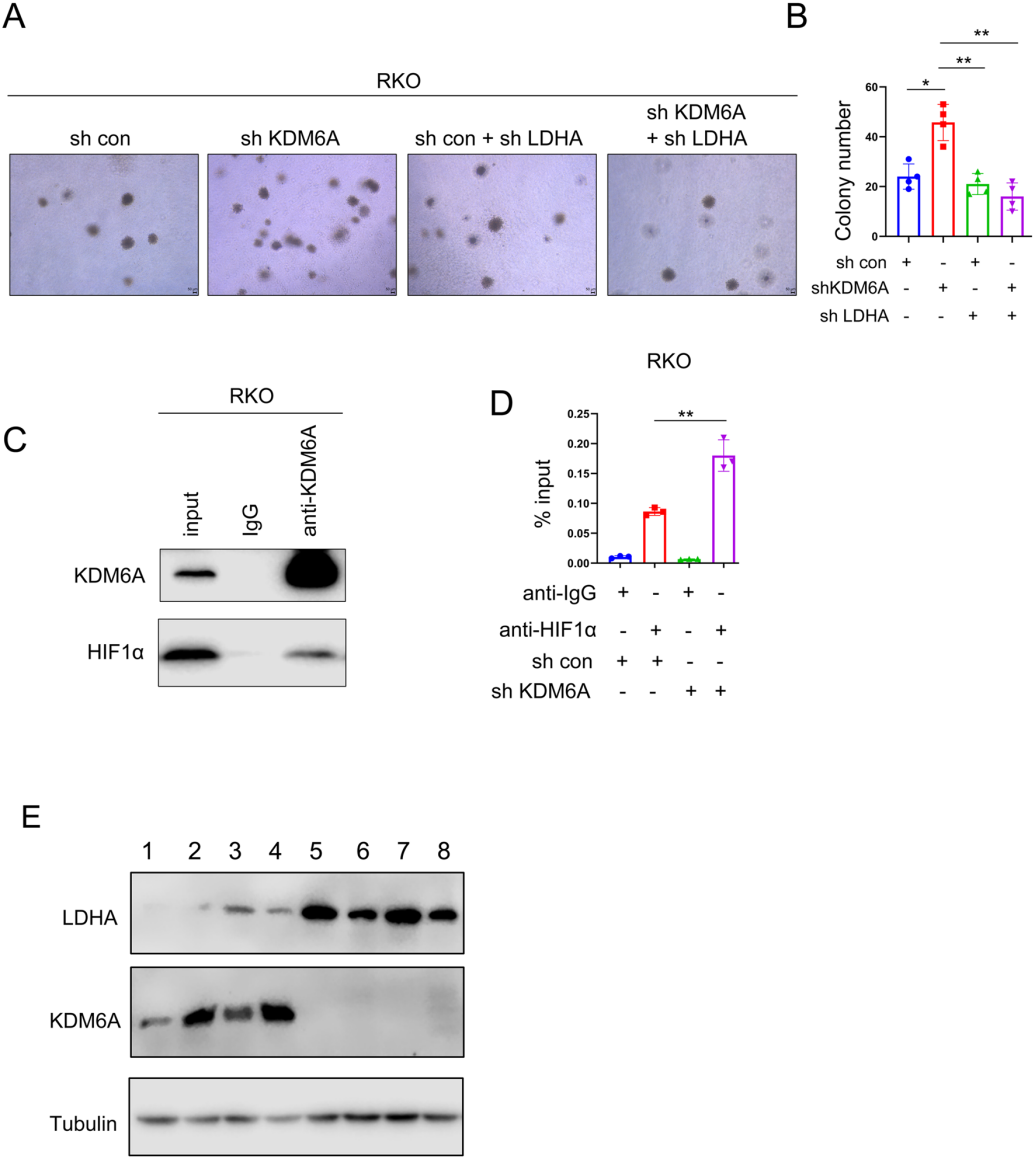
KDM6A inhibits the binding of HIF-1α to the LDHA promoter. **A**, **B** Colony formation assays were used to study the rescue effect of knocking down LDHA on the phenotype caused by knocking down KDM6A, and statistical analysis was performed. Scale bar, 50 µm. **C** The interaction between KDM6A and HIF-1α was detected using immunoprecipitation. **D** Chromatin immunoprecipitation was used to detect the binding of KDM6A to the LDHA promoter. **E** Western blot was performed to examine protein levels of LDHA and KDM6A in colorectal cancer tissues. *, *P* < 0.05; **, *P* < 0.01

To determine whether LDHA mediates the functions of KDM6A downregulation, we further downregulated the expression of LDHA in the cells with knocking down KDM6A expression. The results showed that downregulation of LDHA expression abolished the effects caused by KDM6A downregulation (soft agar colony formation) (Fig. 5A, B). HIF-1α has been reported as an important regulatory factor for LDHA gene expression [20]. We found an interaction between KDM6A and HIF-1α (Fig. 5C). Furthermore, the ChIP assay determined that downregulation of KDM6A expression promoted the binding of HIF-1α to the LDHA promoter (Fig. 5D). In addition, the expression of LDHA was inversely correlated with that of KDM6A in CRC tissues (Fig. 5E).

## Discussion

Although mutations and inactivation of KDM6A have been reported in colorectal cancer [21], there is currently no report on the role of KDM6A in maintaining intestinal epithelial homeostasis and its function in colorectal progression. In this study, we used KDM6A conditional knockout mouse model and found that KDM6A deletion resulted in elongation of villi and crypts in the small intestine, and accelerated the development of AOM/DSS-induced colorectal cancer. These results demonstrated the promoting effect of KDM6A inactivation on the progression of colorectal cancer. In the molecular mechanism study, it was found that KDM6A inactivation upregulates LDHA to promote glycolysis. These findings suggest that reshaping the function of KDM6A may be an important strategy for colorectal cancer treatment.

One of the interesting findings of this study is the illustration of the roles of KDM6A in the progression of colorectal cancer. Previous studies have suggested that inhibiting the demethylase activity of KDM6A can suppress cancer stem cells and inhibit the progression of colorectal cancer [18]. However, these studies were conducted using colorectal cancer cells as models, which have certain limitations. Additionally, previous studies focused on inhibiting the demethylase activity of KDM6A. However, it has been reported that KDM6A can exert its effects independently of its demethylase function [1]. Therefore, it is possible that the inhibitory effect of KDM6A on colorectal cancer development may not rely on its demethylase activity. Consistent with this, in our molecular mechanism study, we found that KDM6A regulates LDHA expression by interacting with HIF-1alpha, promoting glycolysis, and subsequently promoting colorectal cancer progression. This process is achieved by preventing the binding of HIF-1alpha to the LDHA promoter, which is likely independent of the demethylase activity of KDM6A.

Another finding of this study is the role of KDM6A in tumor metabolism. Previous studies have shown that mutations in KDM6A lead to highly active glycolysis in bladder cancer, but the molecular mechanism remains unclear [6]. In this study, we found that KDM6A promotes glycolysis by upregulating the expression of LDHA. Furthermore, the function of KDM6A in colorectal cancer also depends on the expression of LDHA. Therefore, this study elucidates the mechanism by which KDM6A regulates glycolysis, and suggests LDHA as a therapeutic target for colorectal cancer carrying KDM6A mutations.

In summary, this study demonstrated that KDM6A promotes the progression of colorectal cancer by upregulating LDHA and glycolysis. The identification of this offers a promising target for the treatment of colorectal cancer.

## Conclusion

In summary, this study using animal models revealed that KDM6A loss promotes the progression of colorectal cancer through reprogramming the metabolism of the colorectal cancer cells, suggesting that restoring the function of KDM6A is likely to be one of the strategies for colorectal cancer treatment.

### Supplementary Information

**Additional file 1.** The primary data for Western blot.

## Data Availability

All data and materials presented in this study are available in this article.

## Abbreviations

CRC: Colorectal cancer
AOM: Azoxymethane
DSS: Dextran sulfate sodium salt
KDM6A: Lysine demethylase 6A
