# Supplementary figures and images for: Inactivation of KDM6A promotes the progression of colorectal cancer by enhancing the glycolysis
Source: Eur J Med Res. 2024 Jun 6;29:310. doi: 10.1186/s40001-024-01828-1 (PMC11155098; doi:10.1186/s40001-024-01828-1)

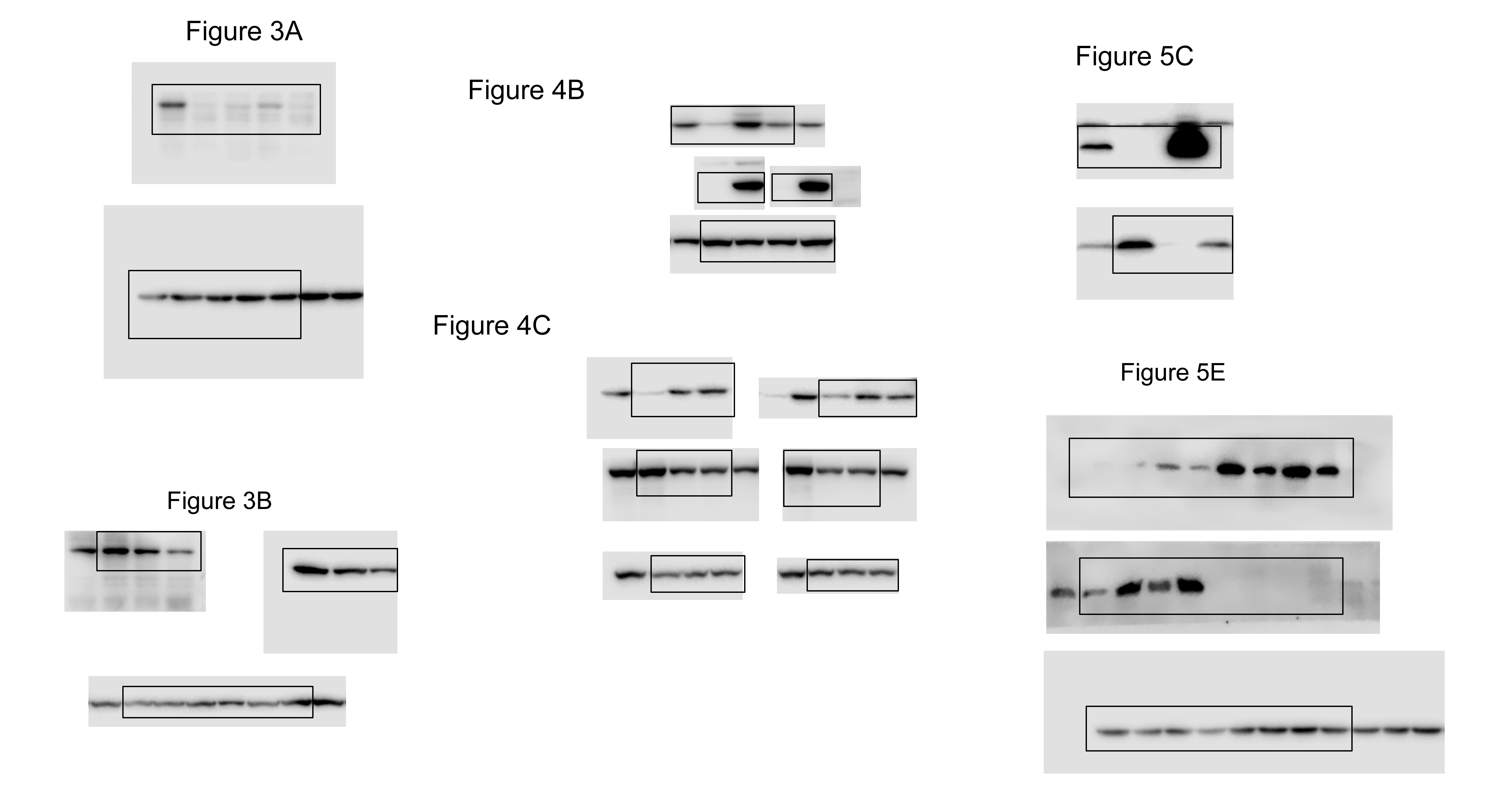

Supplement: Supplementary file 1 — Additional file 1. The primary data for Western blot. [file 40001_2024_1828_MOESM1_ESM.tif]
